# Self-Medication

**Published:** 1911-01

**Authors:** T. B. Rice

**Affiliations:** Greensboro, N. C.


### SELF-MEDICATION.

BY T. B. RICE, PH.G., GREENSBORO, N. C.

Perhaps the greatest evil from a physical standpoint in America to-day is Self-Medication. So let us consider for a few moments some of the causes and suggest some remedy for this growing evil.

I believe the primary cause of this evil arises from a narrow conception of the individual rights of a great number of American citizens, who conclude that they have a God-given right to invade any profession, regardless of personal qualification or fitness, coupled with a burning desire to make money regardless of method.

Many of these men and women who hold this narrow view of American citizenship are shrewd enough to recognize the opportunity afforded them by the superstition and ignorance of millions of our citizens in reference to the science of medicine. Having chosen this field of labor, they proceed to "invent," "discover," or otherwise get in posssesion of some wonderful "cure" for disease—which they proceed to set to music by the aid of printers' ink, with the accent on the dollar. Some of them get their "cures" from "Indian Doctors," others from their "grandmothers," in the good old days when people were honest. Others paid a great sum for their wonderful receipt to some great doctor from a foreign country, whose family had been in possession of it for hundreds of years and is "approved by Grace." Then they proceed to go forth and proclaim their wonderful "cures" to all mankind. And if they can manage to raise a few dollars, our secular, daily and weekly religious papers feel "called" to convey the good tidings to America's sick and explain to them that it is no longer necessary for them to go to the foolish expense of calling in a doctor, and all that is necessary is to buy these wonderful "cures" and keep on buying and taking them.

Many doctors themselves feel "called" to help in this good work by indorsing and prescribing certain head-ache powders and tablets, liver salts, antiseptic solutions, pills, elixirs, etc.

My observations have been that doctors often tell the patient to go to the drug store and buy a bottle of so-and-so and take it according to directions. The patient does so and the next time he feels a little sick he goes straight to the drug store instead of consulting the doctor. He does more, he tells his friends about it and they do likewise.

After the doctors have sufficiently indorsed these remedies to give them a national reputation and made their names household words, the "ethical" manufacturer tells the doctor to go to ——— and advises the consumer through the papers and by letters that they need no longer go to the doctor for a prescription since they have decided to put on the market their great "cure" in convenient packages with full directions, at a price within reach of all.

Then we have thousands of medicine seller swhose knowledge of pharmacy consists chiefly of pouring a ready-made mixture from one bottle to another, together with a full knowledge of the wonderful claims of the nostrum maker and a great desire to help them along by indorsing and making the price attractive by cutting to 19c.

Gentlemen: Is it to be wondered at that the people are doctoring themselves?

WHAT IS THE REMEDY?

I believe that in view of the fact that each state has passed laws regulating the practice of medicine and pharmacy and as these laws were passed primarily for the protection of the people against ignorance and quackery and not for the protection of doctors and druggists, that these laws should be carried out and so amended and construed by the courts as to eliminate every form of quackery. As these laws are now construed, any blacksmith, cobbler or tramp can go forth with his pack of ready-made nostrums, which he makes himself, over-night, at his boarding house, and heal the sick, and be protected by law, if he can raise the money to pay the license exacted, and if he happens to be a veteran of some war, he has a free license to rob the ignorant sick whether he has qualified as a druggist or not, or to practice any profession that requires qualification except law. I believe the lawyers hold that their profession is too sacred for them to enter *without due examination.*

I believe that druggists should practice pharmacy, not medicine, that they should refuse to indorse any patent or proprietary remedy, that they should refuse to allow any nostrum advertised over their names, that they should refuse to distribute booklets setting forth the many wonderful "cures," many of which are indecent and suggestive.

I believe that physicians should practice medicine, not pharmacy, that they should prescribe, not dispense, that they should prescribe drugs, not proprietary remedies, ready prepared under copyrighted names, that the U. S. P. should be their guide, that they should refuse to be "detailed" with copyright remedies or literature on same.

My observations have been that the dispensing physician does not consider so much what his patient needs, as he does what he has on his shelves, that he can supply him, and it often happens that the patient gets not what he needs, but what the doctor happens to have.

I believe that the courts should clearly define the practice of medicine, and what is meant by practice of pharmacy, and that they should deny the right of those who are not duly qualified to practice either or make a pretense thereto.

I do not believe that ready-made or proprietary remedies carrying directions and claiming to cure or mitigate disease should longer be held by the courts to be mere merchandise, as the courts can not regulate the claims made by the manufacturer.

This narrow conception of justice allows the manufacturer, who in many instances is neither a physician nor a druggist to diagnose by mail and practice medicine by wholesale. It allows the seller, regardless of his position in life, moral standard or qualification, to practice both medicine and pharmacy.

I do not believe that Georgia should permit to be sold within her bounds any remedy for the cure, mitigation or prevention of disease, for man or beast, unless same is prepared by a registered druggist, licensed physician or veterinarian, in this or some other state, and then sold only by licensed men of one of the above professions. Nor do I believe that this clear and just interpretation of law would affect the people in any way but beneficially. It would take away from many imaginary rights

It would make room in every community for a competent physician and druggist. It would improve Georgia's health and death rate. It would save the people of the state millions of dollars annually that is now sent away for nostrums and fakes, which is worse than thrown away, provided a just interpretation of our Interstate Commerce Law could ever be reached. I do not believe that it was ever intended to allow public carriers to convey from one state to another any prohibited article. Any article that is prohibited in the state where delivery is made. I believe in state rights. This law denied a state the right to prohibit any article effectually.

